# How to design font size for older adults: A systematic literature review with a mobile device

**DOI:** 10.3389/fpsyg.2022.931646

**Published:** 2022-08-01

**Authors:** Guanhua Hou, Umenwaniri Anicetus, Jingwei He

**Affiliations:** ^1^Pan Tianshou College of Architecture, Art and Design, Ningbo University, Ningbo, China; ^2^School of art and design, Guangzhou University, Guangzhou, China

**Keywords:** age, older adults, smart devices, mobile devices, font size, user experience

## Abstract

With the global society aging, it is necessary to investigate suitable font size based on reading time/speed, readability and legibility for older adults. This study used a systematic review of previous and existing relevant research on font size for older adults and research on the psychophysics of reading and analyzed the outcomes based on reading time/speed, readability, legibility and the usability evaluation methods employed. Studies were selected from databases GOOGLE SCHOLAR, WEB OF SCIENCE, PUBMED, and SCIENCE DIRECT. An inclusion criterion was used to remove duplicates and avoid inconsistencies. Results suggest that older adults preferred larger font sizes. However, there exists a critical size at which readability declines. Inconsistencies in evaluation methods and experimental procedures were observed in the selected articles. This study suggested a reusable catalog of usability evaluation methods, eye tracking for user testing and a questionnaire for inquiry as suitable usability evaluation methods, a uniform metrics to measure font size (visual angle of font) in arcminutes and parameters to consider when investigating font size for older adults to ensure consistency in future studies.

## Introduction

The rapid development of technology over the years has brought about some revolutionary changes, especially in human-computer interaction (HCI). Mobile internet access has become more widely used and comfortable for users (Sohn et al., 2017). The increasing usage of mobile devices by older adults has led to positive outcomes, especially regarding their health and wellbeing. They can now communicate instantly with their doctors/physicians, manage medication, and track fitness. The interface design has evolved and will continue to evolve with time. The past decade has seen a lot of novel design changes to both the hardware and the user interface of mobile devices, e.g., the transition from a feature phone to a smartphone experience. This study focuses on how older adults perceive the font size changes to the mobile user interface by reviewing existing literature. A great deal of research has been done, and even design guideline books have been published to help designers understand users' mental models and how they interact with these interfaces. Since older adults and people generally have varied individual usage scenarios and are affected by age differently, no specific recommendation can serve as a permanent solution. However, an empirical evaluation method and uniform parameters to guide researchers on this topic are suggested. Therefore, it is necessary to have a systematic review of previous studies about font sizes on readability and legibility for older adults to find out the current trend and most favored empirical evaluation method.

Age comes with normative changes (Savage et al., 2019), i.e., as we age, we experience a decline in cognition, vision, and perception. However, age-related changes in cognition are not uniform across all cognitive domains or all older individuals. Attention and memory are the essential cognitive functions most affected by age (Glisky, 2019). These functions are essential in the daily usage of mobile device operations. Although there is a normative cognitive decline with age, enormous variability exists across individuals (Glisky, 2019). Many older people out-perform young adults on some cognitive tasks, and others of the same age do at least as well as the young. For more on changes in cognitive function in human aging, refer to the books “Brain Aging: Models, Methods and Mechanisms” (Glisky, 2019) and “The Handbook of Aging and Cognition” (Craik, 2011).

Visual impairment among older adults is a significant health problem. With advancing age, the normal function of eye tissues decreases, and there is an increased incidence of ocular pathology (Tsai et al., 2005). The decreased visual prowess of affected older adults makes it daunting to see (user interface) UI elements or successfully carry out tasks on mobile devices; this mostly leads to the unwillingness to use smart devices as it is perceived as stressful. Compared to most able youths, the learning ability, adaptability, and acceptance of new technology by the elderly are far inferior; this makes it a hurdle to adapt to the fast-developing technology. For example, older adults find it difficult to use applications on smart devices because of the small sizes of the icons and fonts (Huang et al., 2019). Icons (icon and text) used in modern interface designs are more accessible to younger adults as they have better vision and retention ability to identify and process data faster and more efficiently. This is a crucial problem to consider, as older adults are also an essential part of society, and successful interface design could significantly improve the usability of mobile devices for older adults. More studies should be carried out on the usability of mobile devices for older adults, considering the normative changes that come with age. Many innovative smartwatches help track the wellbeing and fitness of an individual. In the case of industrialized countries, there is much pressure on the health care system, as the older population are more prone to chronic diseases (Ehrler and Lovis, 2014). The use of smartwatches and mobile devices could serve as assistive technologies and support the healthcare system (Ehrler and Lovis, 2014). Aging is also characterized by heterogeneity, i.e., the populations, samples, or results are different. Therefore, It is important to note that older adults should not be generalized as some are computer literate and have at least some experience using mobile devices. As stated earlier, the effect of age is not uniform across individuals. Hence, this study categorized related literature into different age categories for older adults, compared the outcomes and investigated the empirical methods of evaluation used.

Older adults and younger adults exhibit similar cues for evaluating items (Dunlosky and Hertzog, 1997) but provide different judgement of learning patterns due to normative age changes, suggesting that older adults could have a similar response to font size and font style manipulation as younger adults. Mueller et al. (2014) investigated the effect of the processing fluency hypothesis on the judgement of learning (JOL), memory beliefs influence JOLs more than processing fluency. For example, between a 48 point and 18 point font, people's JOLs are higher for the larger font as it is perceived that larger is easier, which leads to a higher JOL (Mueller et al., 2014). Older adults could provide more varied JOLs than younger adults. However, even if there exist similar JOL patterns between older and younger adults, older adults' JOLs are significantly lower than younger adults', given the normative age changes in memory self-efficacy (Cavanaugh and Poon, 1989; Cavanaugh and Green, 1990; Connor et al., 1997). A study by Price et al. (2016) investigating the role of font size and font style on predicted and actual recall performance suggested that older and younger adults predicted a higher recall of large font items than small font items, regardless of font style, and bold style had a higher recall than the regular and italic styles.

## Related works

### A user interface for older adults

User interface design should be simple, easy to learn and able to hold the users' attention for the duration of the specific tasks, imposing less cognitive loads. The user interface consists of well-arranged graphical elements that convey information to the user; however, to make technology accessible to older adults, age-related changes have to be considered. Studies have been done concerning mobile user interfaces for older adults. Kiat and Chen (2015) a study published in “procedia computer science” on mobile instant messaging (MIM) for older adults observed that user interface design features such as small font size, confusing icons and application flow in current MIM applications make them difficult for the elderly to learn and use.

Readability is the arrangement of fonts and words to make written content flow in a simple, easy to read manner. Legibility refers to how easily distinguishable the letters in typesetting or font are from one another. Size plays a massive role in interface design, especially font and icon sizes. Many mobile device companies, have employed relevant design practices to help users select the font size and font style suitable for them; this flexibility allows for a more user-controlled experience. However, future studies should explore more font size options due to the constant increase in the size of mobile displays, which has been evident over the past decade. Research has been done on font size, line spacing and word spacing to determine suitable font sizes for better legibility, readability and reading speed (Epelboim et al., 1994; Rayner et al., 1998; Bernard et al., 2001; Alotaibi, 2007; Wang et al., 2009; Hou and Hu, 2022).

**Figure 1:**
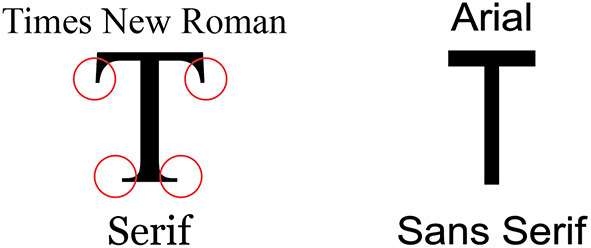
Serif and Sans serif typefaces.

A font typeface is a set of glyphs that have similar designs. Two popularly used font typefaces have been investigated in previous studies i.e., serif and sans serif. A serif typeface has cross-strokes that project from the main stroke of the letter, while the sans serif typefaces do not. A visual reference to the difference between both typeface is shown in Figure 1.

It is essential to note that the defining factor between mobile devices investigated in this study is based on the display sizes, how texts are presented on these displays and the effect of the font sizes on the perception concerning reading speed/time and readability and legibility.

### The font size on mobile devices

Reading smaller font sizes leads to visual fatigue, especially in older adults. Lin et al. (2013) study on the effect of text direction, screen size and character size on legibility and visual fatigue. The stimuli were displayed on two mobile devices: an E-reader to explore visual and visual fatigue and an IPad to explore legibility performance and visual fatigue, Ming typeface (Chinese character). Four different character sizes: 8 pt (2.0 mm ^*^ 2.0 mm), 10 pt (2.7 mm ^*^ 2.7 mm), 12 pt (3.4 mm ^*^ 3.4 mm), and 14 pt (4.1 mm ^*^ 4.1 mm) were used. The screen sizes explored include (6, 8.1 and 9.7 inches). Results suggested that text direction, screen size, and character size significantly affected search time. When the horizontal text, bigger screen, and the 12-pt font are combined, the resultant search time is the shortest. It was observed that, with a fixed screen size, the character size above or below the critical value does not shorten the search time. For the 6 inch and 9.7-inch screens, 12 pt took the shortest search time, and 12 pt and 14 pt were suitable for the 8.1-inch screen size. The 12 pt and 14pt were suitable for the E-reader on the 9-inch screen. Too small fonts are the main cause; search time significantly increased when 8 pt texts were read. Therefore, when a smaller font is read, the user's eyes get tired, leading to visual fatigue.

Larger font sizes prove optimal for standard reading scenarios. A study by Wang et al. (2009) investigated the effects of inter-line spacing and inter-character spacing on performance and perceived text readability. The stimuli (Chinese characters) were presented on NEC mobile phones. The effect on visual fatigue and preference was investigated on fixed font size (8 points). It was observed that increasing both the Chinese inter-character spacing and inter-line spacing significantly improved reading performance for older adults. In a study by Hou et al. (2020) investigating the effect of Chinese text spacing and size on older user experience, the effects on usability, visual comfort, cognitive load and reading performance were explored. An eye tracker was used to obtain eye movement data which was then analyzed. It was observed that usability improved with an increase in font size. Older adults (59–79 years) preferred larger font size of (10.5–15pt) and spacing (0.5–1.0pt) in terms of readability, usability, visual comfort and reading performance on an iPhone 6s and found larger sizes than mentioned unsatisfactory (Hou et al., 2020). These sizes were optimal for standard reading scenarios, visual search or skimming through a webpage with articles or paragraphs.

Font typeface affects the readability of characters or texts in a passage, irrespective of the medium. In a study by Chatrangsan and Petrie (2019) on the effect of typeface and font size on reading text on a tablet computer, the stimuli were displayed on a 4^th^ generation iPad tablet running IOS and safari in Thailand and the UK. Three font sizes (14, 16, 18 points) were explored with two typefaces, serif and sans serif. It was observed that older participants were less fatigued during the skim reading task than during the detailed reading. In both countries, reading time was significantly affected by font size, not by typeface 0.18 pt was significantly faster to read than 14 or 16 points. Comprehension was significantly better with larger and serif fonts in both countries. Participants in the UK found the sans serif typeface easier and less tiring to read, while Thai participants found the serif font easier and less tiring to read. On overall preference, more than 50% of the UK participants chose 18 points sans serif, whereas more than 50% of Thai participants chose 18 points. As is evident in previous studies, larger font/character sizes are suitable for older adults in terms of readability, legibility, faster reading time and reduced visual fatigue.

Studies have been done to determine if un-spaced and spaced texts significantly affected readability and reading speed. Epelboim et al. (1994) suggested that it was easy to read un-spaced texts as they had a shorter saccade length. Results suggested that subjects read un-spaced texts with the same level of comprehension and percentage of regression as they read spaced text, but apparent vision prowess was essential. However, a contradicting study by Rayner et al. (1998) argued the legibility and readability of un-spaced text. Instead, the study suggested that it was difficult to read an un-spaced text for most readers. This study directly argued with the study results (Epelboim et al., 1994) as primarily based on one subject. Epelboim et al. (1994) also stated the need for clear vision, which is not the case for some older adults as age-related changes in vision significantly affect the ability to read and understand un-spaced texts efficiently, thus, inducing cognitive load leading to unwillingness on the part of the individual (Hou et al., 2020). His study observed that usability decreased as the word spacing increased but improved as the line spacing increased. However, upper bounds of WS and LS for visual comfort were observed when the standard WS increased by 0.7 pt, and LS was 1.2 times the standard. Mobile device companies have employed relevant design practices to help users select the most suitable font size. This flexibility allows for a more user-controlled experience on mobile devices. Studies on font size and spacing suggest that older adults prefer larger font sizes and find the spaced text more legible and readable.

Visual limitations significantly influence the readability of texts irrespective of the font size. Yeh (2015) examined the performance of younger and older users (65+ years) when reading text messages on mobile devices and took account of their visual limitations. The stimuli were displayed on a 9.7-inch tablet at a viewing distance of 40 cm, with five font sizes (6, 8, 10, 12 and 14 points) and exposure time (0.2, 0.4, 0.6, 0.8, and 1.0 s) with Arial typeface and reading time was evaluated. The younger adults read 12 points accurately, whereas the older adult found it challenging to read even at 14 points font size. This could be a result of visual ability deterioration caused by aging. Also, the significant difference between the younger adults and older adults in this study, where younger adults read text messages correctly 87.74% of the time at 0.4 seconds exposure time as opposed to the 39.87% of the older adult, could be different in a scenario involving only older adults as this will provide results with high significance to older adults of different age range. Another study by Yeh (2020) on the effect of touchscreen button position and font size on a touch screen for older users (at least 65 years). The stimuli were displayed on a 9.7-inch tablet at a viewing distance of 40 cm. It was observed that a top positioned button with a font size of 22 points optimized the performance of older participants as opposed to the font size of 10 points and a bottom positioned button, which degraded their performance.

Age significantly affects reading time and viewing distance. In a study by Lege et al. (2013) on the effects of age on the readability of characters on e-book terminals, the reading time and viewing distance were investigated. The stimuli (Japanese characters) were displayed on third-generation IPad3, IPad2 and regular paper. Three character sizes (small 8pt, medium 10.5pt and large 18pt) were displayed on the IPad3 and paper, while the IPad2 was used for medium size. Reading deteriorated for older adults with more minor characters. Though larger characters slightly improved readability for the elderly, reading speed remained slow even with 18 pt characters. When the character size was small, the younger subjects shortened the viewing distance to maintain the viewing angle; however, the rate of shortening the viewing distance was less in senior subjects. It was suggested that presbyopia might have affected the elderly subjects. In the subjective evaluations, the younger subjects rated the size (10.5pt) as most comfortable to read, while the middle-aged adults stated the size 18 pt as more accessible (Lege et al., 2013). As the character becomes larger, the easier its readability for older subjects. Thus, larger size characters appear more suitable for senior individuals.

Viewing distance increases with the increase in character size. In a study by Darroch et al. (2005) on the effect of age and font size on reading text on a handheld device, an HP IPAQ was used to present the stimuli to the participants (51–78 years). The device was held at a comfortable distance, subjective to the subjects. A font size range of 8–12 points was recommended for older adults using smartphones for reading accuracy with a typeface Microsoft san serif font. A study by Hasegawa et al. (2006) investigated the aging effect on the visibility of graphic text on mobile phones using Japanese characters. The stimuli were presented on an LCD of 260 k color TFT to the participants (60–79 years) at a viewing distance of 30 cm. It was observed that reading performance deteriorated as the size of the characters became smaller and as subjects became older. Visual acuity and cloudiness were strongly correlated with age and affected reading performance. However, age had a higher correlation with reading speed than visual acuity and cloudiness. Moreover, as subjects became older, the viewing distance became shorter, and the character size smaller.

These studies have provided concrete theoretical and experimental evidence of how fonts are perceived and why older adults prefer large fonts in terms of reading performance. A solid observation from the literature review is how varied the variables of the experiments are, the methods used in these experiments and the participants make up. The primary purpose of this paper at its base is to bring focus to older adults only in terms of design innovations. We can confidently bring an argument between studies with two groups of participant make-up (i.e., younger and older adults) and the studies with only older adults. Although they mainly arrived at a consensus that larger font sizes improved performance for older adults, there exists the matter of subject comparison. Comparing older adults with younger adults resulted in an apparent significant performance difference. However, performing a design usability study (in this case, font size) among older adults will produce more meaningful information on the challenges faced by the elderly when reading a font, giving designers and researchers alike a first-person perspective on the thought process and JOL patterns employed by the elderly when evaluating font sizes, font style or font typeface.

The contribution of this study is to provide font size recommendations for age range and use case scenarios based on existing literature, and most importantly, detailed and uniform parameters that we deem necessary for conducting research on older adults' perception of font size in terms of reading speed/time, readability and legibility. With this contribution, we believe that future researchers will be able to find related articles uniformly and with ease and find innovative research gaps not just for the elderly but also for the general user.

#### Research questions

In order to have a logical flow in the data extraction from previous related studies, some research questions have been developed to systematically gather enough evidence to support the contribution of this study. Five (6) research questions were defined to accomplish this study's goal. These research questions are essential in researching font sizes for older adults as they will help identify relevant literature in this area for extensive reviewing. Their motivations are shown in Table 1.

**Table 1:** Research questions.

**No**.	**Research question**	**Motivation**
RQ1	What publication channels are the primary targets for the readability and legibility of font sizes for older adults?	To determine the different sources of related studies that have been published. This information will help researchers know some of the journals with articles that investigate this area of research.
RQ2	What are the current font sizes recommended by existing studies, and for what age range and use case scenario?	To categorize the font size recommendations in selected articles according to the age range of older adults, use case scenarios and compare outcomes.
RQ3	What is the metric system of measurement used for font sizes?	To provide information on the metric system used for measurement and suggest a uniform metric for future studies.
RQ4	Which empirical methods are used to evaluate reading time/speed, readability and legibility of font size?	To recommend a uniform method for evaluating the readability and legibility of font sizes for older adults.
RQ5	What are the parameters used in evaluating font sizes for older adults?	To examine parameters used to evaluate font sizes across selected articles and recommend a uniform parameter set for future studies to provide.
RQ6	What is the age range of the subjects in the study?	To extract specific age of the subjects to aid in the font size recommendation with respect to age range.

#### Logical relationship

For a better understanding of the logic in this study, a logical relationship between the search string, quality assessment of the articles and research question is provided. The logical relationship of this study is shown in Table 2.

**Table 2:** Logical relationship.

**Search strategy**	**Quality assessment**	**Research questions**
**(Table 3)**	**(Table 4)**	**(Table 5)**
Article source	QA6	RQ1
Display context	QA1, QA2	RQ5
User interface	QA8	RQ2, RQ3
Usability topic	QA3, QA4, QA5	RQ4
Age	QA7	RQ6

## Systematic literature review methodology

A systematic literature review (SLR) entails evaluating and interpreting all available research relevant to a particular research question or topic area, or phenomenon of interest (Kitchenham, 2004). With the phenomenon of interest being “font-size reading speed/time, readability and legibility for older adults,” data collected for this SLR will be thoroughly screened for relevancy to the topic.

### Search strategy

The search strategy employed involves the selection of resources and the identification of search terms. A set of search automated search engines from the most relevant sources in human-computer interaction, computer science and health were selected to perform the search for papers: GOOGLE SCHOLAR, WEB OF SCIENCE, PUBMED and SCIENCE DIRECT. The search string was determined using the PICO criteria (Stone, 2002; Methley et al., 2014): population, intervention, comparison and outcome. The selected search string should provide maximum coverage but manageable size (Zapata et al., 2015).

The terms used, which are based on the research questions, have been selected by using four different scopes as a starting point: (1) Display context, which includes mobile displays and computer displays as the target; (2) the element of a user interface considered in this study, i.e., fonts sizes; (3) the reading time, readability and legibility characteristics of the font size; (4) the age, taking the older adults as the specific area of concentration for this study. The Boolean operator OR is used to join alternate terms, and the AND is used to join two major parts. The search string is shown in Table 3.

**Table 3:** Search string.

**Scope**	**String**
Display context	(Smartphone OR touchscreen OR mobile device OR tablet) AND
User interface element	(Font* size*) AND
Usability topic	(readability AND legibility) OR (readability OR legibility) OR (reading speed OR reading time) AND
Age	(Older adults OR elderly OR age OR aging OR senior OR older readers)

### Inclusion and exclusion criteria

Each study recruited from the initial search process was evaluated through an inclusion criteria (IC) and exclusion criteria (EC) to determine whether or not it should be admitted as one of the selected studies. The inclusion criteria used for the selection are as follows:

**IC1**. The paper focused on font size for mobile devices, smartphones, tablet devices or Phablets; this includes mobile devices simulated on computer monitors.
**IC2**. The paper focused on older adults, older readers, or comparing both older and younger adults.
**IC3**. The paper investigated font size performance in reading speed/time, readability and legibility.
**IC4**. The paper presents empirical results.
**IC5**. The paper details the experiment design and methods.
**IC6**. The focus lies in the older adult's perception of font sizes regarding reading speed/time, readability and legibility.
The paper that conformed to at least one of the following criteria was excluded:
**EC1**. The paper focused on younger adults alone.
**EC2**. The paper was published before 2000. The first touchscreen phone, marketed as a smartphone, was re-leased by Ericsson in 2000 (Lobo and Kaskaloglu, 2011). This applies to articles involving smartphones with touch capability.
**EC3**. The devices' interaction modalities (i.e., touch screen) are not investigated in this study.

### Quality assessment

This quality assessment aims to weigh the importance of the selected articles when results are discussed and to guide the interpretation of findings (Kitchenham, 2004). The quality assessment checklist is shown in Table 4. The quality assessment in this study was inspired by a previous mapping study (Ouhbi et al., 2015).

**Table 4:** Quality assessment checklist.

**No**.	**Quality assessment question**	**Answer**
QA1	Is the experimental instrument/technology used detailed in the article?	(+1) Yes/(+0) no/(+0.5) partially
QA2	Are the stimuli displayed on a computer screen or mobile screen	(+1) Mobile/(+0.5) computer
QA3	Is the readability and legibility evaluation method specified in the article?	(+1) Yes/(+0) no
QA4	Our empirical results of the readability and legibility evaluation shown?	(+1) Yes/(+0) no
QA5	Does the article discuss any findings of the readability and legibility evaluation?	(+1) Yes/(+0) no
QA6	Has the study been published in a recognized and stable publication source	(+2) Journals/(+1) conferences
Q7	Whether the research objects of the article are the elderly or whether the article involves comparison between the elderly and the young?	(+1) Yes/(+0) no
Q8	Does the article state the font size and metric system of measurement used for font sizes?	(+1) Yes/(+0) no

**QA1** scores partially when the technology is not detailed in the article but can be deduced by screenshots provided. **QA6** is rated by analyzing the conference proceeding and Journal Citation Report (JCR) (resurchify, 2015; Clarivate, 2020). Journals and conferences score differently in **QA6** because the average paper in a top journal is more polished than the average paper in a top conference (Bowyer, 2012). Although the acceptance rate of a conference and a journal should not be compared, it is more challenging to publish in a top journal than in other publication channels (Bowyer, 2012). The importance of a paper is determined when the **QA** score is above **5 points**. The maximum score an article can obtain after complying with the eight quality assessment questions criteria is **9 points**.

### Data collection process

The data collection process was based on the research questions, and the relevant information presented in Table 5 is therefore extracted to answer them. The process was carried out by completing a data form.

**Table 5:** Data extraction.

**No**.	**Extracted data**
RQ1	Publication source should be extracted to answer this question
RQ2	The preferred font sizes recommended and the reason for the results in the publication should be analyzed to answer this question.
RQ3	The metric system used to measure the font size should be analyzed.
RQ4	The methods and process of evaluation should be extracted to investigate how the readability and legibility evaluations are carried out; method, duration and number of participants.
RQ5	Publication evaluation methods should be investigated to obtain the parameters and factors considered; viewing distance, screen elevation, glare, display type, and experiment environment.
RQ6	The specific age of subjects should be collected.

## Results

This section describes the results obtained to answer the research questions in Table 1.

### Study selection

**Figure 2:**
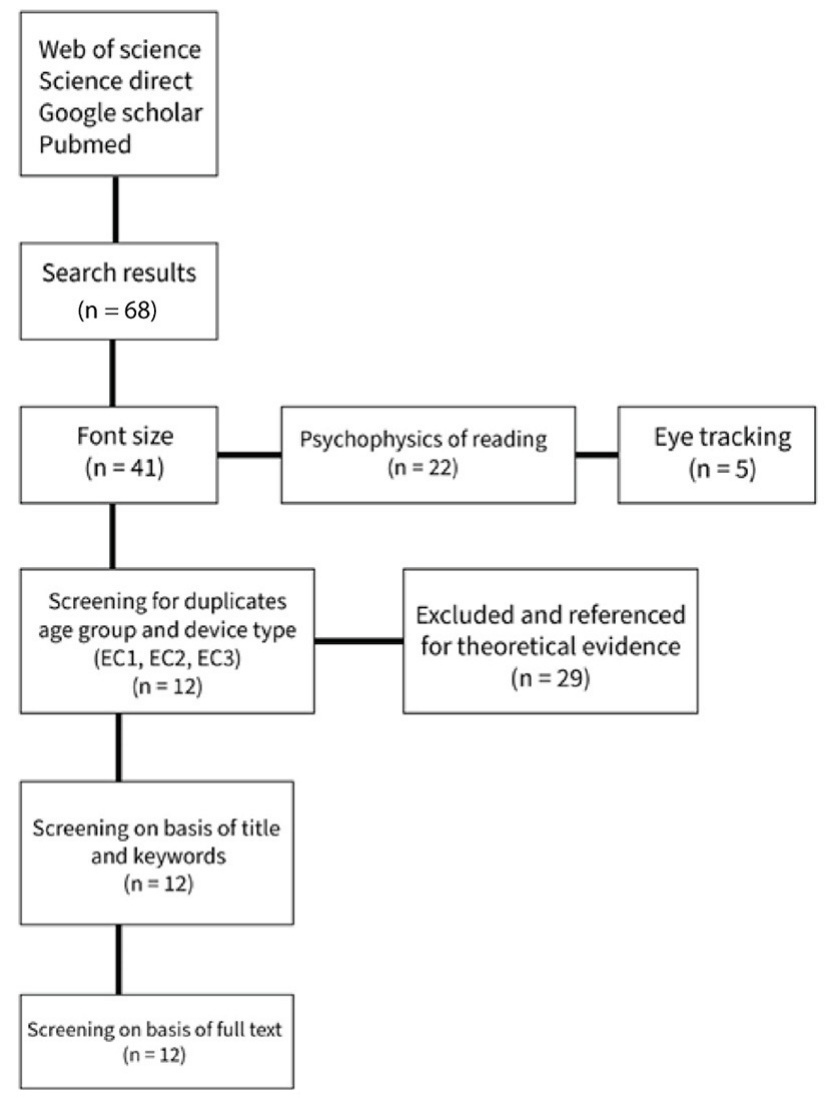
PRISMA flow diagram.

The study selection process took place in April 2021. The databases “GOOGLE SCHOLAR, WEB OF SCIENCE, PUBMED and SCIENCE DIRECT” were the main search engines used to perform this review's literature search. A total of 68 papers were obtained in the search phase, of which 22 papers are on the psychosis of reading which serves as evidence for our suggestions in the discussion section, 41 papers on font size, and five papers on eye-tracking. After screening the 41 papers on font size with Inclusion and exclusion criteria, 29 papers were excluded for not meeting one or more inclusion criteria or meeting one or more exclusion criteria set for this study. However, some of the excluded papers were referenced for theoretical evidence where relevant. The remaining 12 papers were screened based on keywords and relevancy to this study. The full texts of these 12 papers were investigated and finally selected for this review, as shown in Figure 2. Table 6 presents a list of the papers eventually selected and their QA results.

**RQ1. What publication channels are the primary targets for the readability and legibility of font sizes for older adults?**

**Table 6:** Papers selected and quality assessment results.

**Author**	**Pub. Year**	**Pub. channel**	**Pub. name**	**Ranking**	**Quality assessment**								
					**1**	**2**	**3**	**4**	**5**	**6**	**7**	**8**	**Score**
Darroch et al., 2005	2005	C	Lecture notes in computer science (including subseries lecture notes in artificial intelligence and lecture notes in bioinformatics)	Q2; Q3	1	1	1	1	1	1	1	1	8
Hasegawa et al., 2006	2006	J	Gerontechnology	Q4	1	1	1	1	1	2	1	0	8
Fujikake et al., 2007	2007	C	Lecture notes in computer science (including subseries lecture notes in artificial intelligence and lecture notes in bioinformatics)	Q2; Q3	1	0.5	1	1	1	0.5	1	1	7
Wang et al., 2009	2009	J	Educational gerontology	Q3	1	1	1	1	1	2	1	1	9
Hasegawa et al., 2009	2009	C	Lecture notes in computer science (including subseries lecture notes in artificial intelligence and lecture notes in bioinformatics)	Q2; Q3	1	1	1	1	1	0.5	1	0	6.5
Lege et al., 2013	2013	C	Lecture notes in computer science (including subseries lecture notes in artificial intelligence and lecture notes in bioinformatics)	Q2;Q3	1	1	1	1	1	1	1	1	8
Chatrangsan and Petrie, 2019	2019	J	Proceedings of the 16th web for all 2019 personalization - personalizing the web, W4A 2019		1	1	1	1	1	1	1	1	8
Ziefle, 2010	2010	J	Applied ergonomics	Q1	1	1	1	1	1	2	1	1	9
Kong et al., 2011	2011	J	Ergonomics	Q2	1	0.5	1	1	1	2	1	1	8.5
Yeh, 2015	2015	J	Perceptual and motor skills	Q4	1	1	1	1	1	2	1	1	9
Yeh, 2020	2020	J	Heliyon	Q1	1	1	1	1	1	2	1	1	9
Hou et al., 2020	2020	J	Aging and society	Q2	1	1	1	1	1	2	1	1	9

*Pub., Stands for publication*.

The publication channels are varied since there are 10 different publication sources in the 12 papers. The only repeated source is the “Lecture Notes in Computer Science (including subseries Lecture Notes in Artificial Intelligence and Lecture Notes in Bioinformatics)” conference proceedings, although the three articles were published in different years: (Darroch et al., 2005; Fujikake et al., 2007; Hasegawa et al., 2009). The 10 publication sources are distributed into four conference proceedings and eight journals.

The publication sources are mostly (Applied) Ergonomics, computer systems and gerontology (the study of old age, the process of aging and problems faced by older people).

The ranking of their publication sources can measure the papers' reputation. Of the eight journals of publication in the selected articles, three are ranked as Q1 in the JCR ranking if the best rank for the journal is considered. Concerning conferences, 3 out of 4 are ranked Q2; Q3 (resurchify, 2015; Scimago, 2019). One of them is ranked in the top-level, A+: SIGCHI Conference on Human Factors in Computing Systems (Bernard et al., 2001).

**Figure 3:**
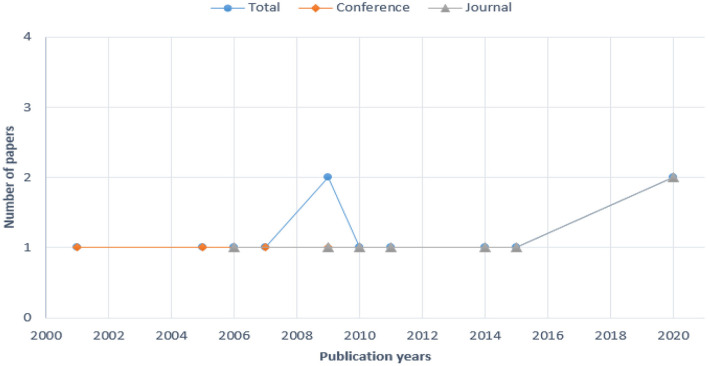
Number of papers per year.

The papers selected were published between 2001 and 2020. Figure 3 shows the number of papers published per year; 1 in 2001, 1 in 2005, 1 in 2006, 1 in 2007, 2 in 2009, 1 in 2010, 1 in 2011, 1 in 2014, 1 in 2015 and 2 in 2020.

**RQ2. What are the current font sizes recommended by previous studies, and for what age range and use case scenario?**

Some of the papers selected provided some kind of font size recommendation for older adults. Five papers provided font size recommendations for mobile devices in varied reading scenarios. The legibility of print depends on the physical characteristics of the text and viewing conditions and the vision status of the reader (Legge and Bigelow, 2011). Therefore, data for the viewing conditions such as viewing distance, experiment atmosphere, stimuli and visual acuity of the reader need to be noted as they significantly affect the font size and visual angle of a font. Table 7 provides detailed information on the font size recommendations and scenarios extracted from selected papers.

**Table 7:** Font size recommendations from selected paper and scenarios.

**Author**	**Age range**	**Participant no**.	**Vision status**	**Device**	**Resolution /size**	**Font style**	**Recommended font size**	**Scenario**
(Darroch et al., 2005)	51–78 years	12	All participants had 20/40 vision or better at a distance of 40.6 cm.	LCD of 260 K color TFT and 132 × 176 dots	640 x 480 px	Microsoft Sans Serif font	A range of 8–12 points to maximize reading for reading tasks	The participants held the device at preferred viewing distances for the reading task.
(Wang et al., 2009)	Mean age 66 years	12	All the older adults had normal vision or corrected to normal vision.	NEC mobile phones (NEC N6305)	480 x 320 px	Chinese characters	8 Points	For reading tasks with Chinese inter-character spacing
(Ziefle, 2010)	55–73 years	40	Null	Siemens S45	101 x 80 px	Arial	8 points (font size) and 12 points (preview size)	For menu navigation on small screen devices. When the preview size was more significant than the font size, readability performance improved menu selection.
(Lege et al., 2013)	60–89 years	28	Subjects who usually wore glasses for reading were allowed to use them for the experiment.	IPad 1,2,3	9.7 inch	Japanese characters	18 point	For reading tasks with Japanese characters.
(Yeh, 2015)	65 years and older	Null	All participants reported 16/20 corrected visual acuity or better.	Tablet	9.7-Inch	Arial	14 Point (42 arcminutes)	For reading tasks on mobile devices Viewing distance = 40 cm Screen elevation = 30 degrees Glare = none Desk to screen = 23 cm
(Chatrangsan and Petrie, 2019)	62–84 years	36	Subjects who usually wore glasses for reading were allowed to use them for the experiment.	Ipad	9.7 inch	Arial and Times new roman (Serif and san serif)	18 point	For reading task
(Yeh, 2020)	65 years	32	All participants had an adequate vision, or vision corrected to above 0.8 and lacked any significant eye condition (e.g., color blindness, amblyopia, or blindness).	Tablet	9.7-Inch	Arial	22 Points (66 arcminutes)	For reading tasks on mobile devices display size = 9.7 inch Viewing distance = 40 cm Screen elevation = 30 degrees. Glare = no The tablet was placed on a 70 cm high table with the center of the screen 23 cm distant from the desk.
(Hou et al., 2020)	57–70 years	20	The tests were conducted with the participants' daily visual aids.	IPhone 6, 5.5'	1920 px * 1080 px	Chinese characters	14 px for information search 17 px for intensive reading 17–20 px for reading news, novels and instruction/ manuals	For reading tasks on mobile devices with Chinese characters. Viewing distance = (held at a comfortable distance)

**Figure 4:**
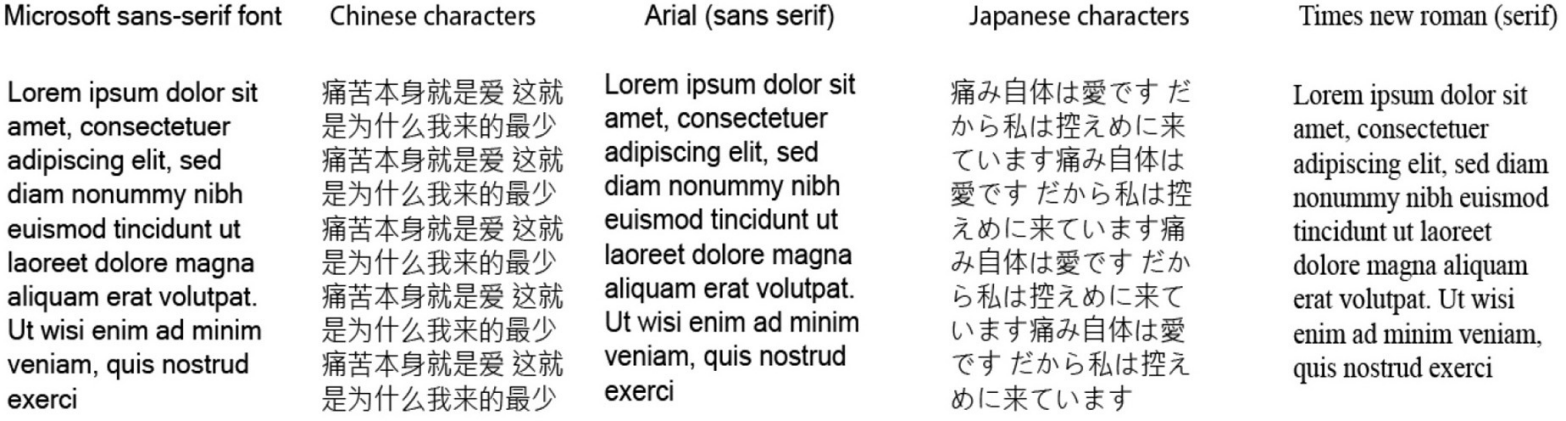
Specific fonts and font style as detailed in Table 7.

In order to easily identify the font styles detailed in Table 7, a visual reference is provided as Figure 4.

The use case scenario was generally consistent across all selected papers (i.e., reading from the device screen) except for the study by Ziefle (2010), in which the use case scenario was focused on menu navigation on small screens. All the studies detailed varied viewing distances, with some allowing the participants to hold the device at comfortable reading distances and with their daily reading aids. The average age for the older participants across all studies was 70 years, with the least and highest ages being 55 and 83, respectively.

In one reading scenario, (Fujikake et al., 2007) investigated the readability of Japanese texts on mobile phones' LCDs with a set illuminance. However, the effect of the illuminance was not discussed in detail. The results suggested that readability deteriorates significantly as the characters become smaller. It was also observed that readability was higher with higher contrast displays. In a study by Hou et al. (2020) on Chinese characters, the results suggested that older adults preferred the character size 14px for information search, 17px for intensive reading, and 17–20px for reading news articles, novels and instruction manuals.

The lighting in the experiment environment is an essential factor that affects productivity. Some studies simulated environmental lighting in their experiment to observe the effect on reading time/speed, readability and legibility of font sizes (Yeh, 2020) performed their experiment in a classroom to minimize outside inference, sunlight was adequate, and minimal noise and a controlled temperature of 26 degrees C. Another study by Hou et al. (2020) simulated ambient lighting using 40-Watt LED lights during the experiment. Although the direct effect of the environmental factors was not explored, we can conclude that the experiment's environment influences the size recommendations in these studies. Ko et al. (2014) experimented with a matte surface LCD screen and an external glare source. However, there was no significant effect of glare on readability; glare had a significant effect on viewing distance as participants reduced their viewing distances to eliminate the glare. It is important to note that the environment and environmental lighting influence the productivity of an individual. Therefore, researchers should consider and provide information on the environmental condition and lighting for the experiment. This information can be collected in a questionnaire specifically made to evaluate how the subject feels about the simulated environment.

The legibility of print depends on the physical characteristics of the text and task demands, viewing conditions and the vision status of the reader (Legge and Bigelow, 2011). The findings in this review point to the result that older adults prefer larger font sizes in terms of reading time/speed, readability and legibility. Reading speed in words per minute was introduced as a metric for measuring legibility (Tinker, 1963). There is a critical print size below which reading speed (i.e., legibility) declines sharply (0.15°/9–0.3°/18 arcminutes). This is dependent on the individual, stimulus factors such as font (Mansfield et al., 1996) and methods of evaluation of critical print size (Legge and Bigelow, 2011). Reading speed declines for extremely large print (characters subtending more than 1°/60–3°/180 arcminutes) (Legge et al., 1985; Susana et al., 1998). However, this decline is not as sharp as the critical print size. Therefore, bigger may not always mean better font sizes for older adults.

From the data extracted in Table 7, some studies experimented with fixed viewing distance. A headrest was used to keep the participant's heads in place throughout the experiment to prevent the participants' involuntary head movements from altering or significantly influencing the results. The viewing distance is an essential factor when investigating suitable font sizes. In order to obtain the visual angle of the font in arcminutes, the viewing distance is essential. Some papers accounted for a glare source, illuminance produced by the display or light source in the experiment room. All papers performed an initial visual acuity test on participants before the experiment to avoid any inconsistencies in the results. Yeh (2020) included a visual acuity range of “adequate vision, or vision corrected to above 0.8 and lacking any major eye condition (e.g., color blindness, amblyopia, or blindness)” as an inclusion criterion to ensure a consistent result.

**RQ3. What metric system is used to measure the font size?**

Some of the papers selected in this review gave font size recommendations. Although the metric systems used were inconsistent, the most used metric is “points.” Eight papers used points for the font sizes, two papers used pixels, one paper used millimeters, and one provided the visual angle in arcminutes of the font size recommended in their study. This study calculated and recorded the visual angle in arcminutes for the selected papers that provided the font size and viewing distance data in Table 7. For font size, we recommend using arcminutes as the metric system. The reasons will be explained in detail in the discussion section.

**RQ4. Which empirical methods are used to evaluate reading time/speed, readability and legibility of font size?**

The method most frequently used to evaluate the reading speed/time, readability and legibility of font size is that of a questionnaire. The questionnaire consists of a set of questions participants answer after the tasks/ experiment in the study. The questionnaires were used to get the participant's opinions on the font size they preferred or disliked by having participants rate the font sizes used in the experiment. Four papers used the think-aloud method, with one of the evaluations that worked with questionnaires also supported by the “think out loud” method. The “think out loud” method involves participants thinking aloud (i.e., reporting back after stimuli disappear) as they perform a set of specified tasks. Participants were asked to say whatever came into their minds as they completed the task. One paper did not provide information on the type of evaluation method used in the study (Hasegawa et al., 2009).

One (Hou et al., 2020) study evaluated the readability and legibility of font size and word spacing using an eye-tracking device (Dikablis). The blink rate was recorded with an eye tracker, and the blink duration was divided by the total time of the experiment (40 min). The higher the blink rate, the longer the time the eye will be closed, triggering declines in usability and visual comfort (Hou et al., 2020). The result obtained suggests that the pupil area and blink rate have significant correlations with elements of user experience, i.e., if the font size used in the stimuli results in more blink rate, the font size is said to be unsuitable or cause fatigue to the reader.

**RQ5. What are the parameters used in evaluating font sizes for older adults?**

The selected studies evaluated reading speed/time, readability and legibility using specified parameters; viewing distance, screen elevation/inclination, glare, font type and display type. Detailed information is presented in Table 7. Five studies investigated different font types, display types and sizes, respectively. Two of the five studies investigated Chinese characters, Korean characters, and Japanese characters. Three papers investigated varied viewing distances, with only two investigating fixed viewing distances. Three studies provided the angle of elevation/inclination of the display used to present the experiment's stimuli. One of the studies allowed participants to hold the device at a comfortable distance for the experiment. The viewing distance used in the two studies was not recorded. Therefore, the visual angle in (arcminutes) of the recommended fonts in these two studies cannot be calculated. One study also investigated the readability and legibility of font size for older adults with an external glare. However, results suggested that glare had no significant effect on readability and legibility of font size but significantly affected viewing distance as participants adjusted their position or the device to eliminate the glare on display (Ko et al., 2014).

**RQ6. What is the age range of the subjects in the study?**

In order to satisfy this research question, the articles selected in this study included only older adults or a comparison of both older and younger adults as the subjects in the research. This research question corresponds to the quality assessment obtained in QA7. The specific age range in these articles are extracted in Table 8.

**Table 8:** Subjects ages.

**Article**	**Total participants**	**Older subjects age (years)**	**Middle aged subjects (years)**	**Younger subjects age (years)**
Darroch et al., 2005	24	18–29	-	61–78
Hasegawa et al., 2006	88	60–79	40–59	20–79
Fujikake et al., 2007	Experiment 1 = 78 Experiment 2 = 98 Experiment 3 = 120	Experiment 1. mean = 39.9 ± 17.1 Experiment 2. mean = 44.5 ± 18.5 Experiment 3. mean = 46.9 ± 18.6		
Wang et al., 2009	12	Mean = 66	-	-
Hasegawa et al., 2009	Experiment 1 = 30 Experiment 2 = 64	Experiment 1 = 19–23 Experiment 2 = 19–76		
Lege et al., 2013	112	60–89	30–59	17–29
Chatrangsan and Petrie, 2019	54	62–84	-	18–23
Ziefle, 2010	40	55–73	-	-
Kong et al., 2011	20	Mean = 66.9	-	Mean = 24.9
Yeh, 2015	62	Mean = 27.7	-	Mean = 68.4
Yeh, 2020	64	18–35	-	65
Hou et al., 2020	190	59–79	-	-

## Discussion

In light of the findings observed from the systematic literature review, it is evident that vital information is backed by evidence on suitable font sizes for older adults for a mobile device. However, this paper focuses on the methods by which these sizes are evaluated. The main contribution of this paper is to provide a uniform method to evaluate font size by systematically reviewing relevant articles. This contribution is discussed under four subheadings. The first goal is to provide font size recommendations for older adults, age range, and use case scenarios from the reviewed articles. Secondly, to determine a uniform metric system of measurement for font sizes. Thirdly, to suggest a usability evaluation method for font size evaluation and how to implement it for older adults. Lastly, to provide uniform reusable parameters to factor in when conducting an assessment experiment for font size. These suggestions are backed by an extensive literature review and aim to provide consistency among related studies and speed up information search for future researchers and designers.

### Font size recommendation for older adults

The font size recommendations made in this study result from the review of existing literature on mobile devices, from feature phones to smartphones to tablets (like the iPad), with specific resolution and age range over the past decade. These recommendations provide a concrete overview for designers to understand what font size is suitable for specific scenarios, the devices in which these sizes can be employed and the age range of the older adults with corresponding font size range. In the usability of font size for older adults, there is very substantial evidence that older adults prefer larger font size irrespective of device display resolution, device type or font typeface (Darroch et al., 2005; Huang et al., 2009; Wang et al., 2009; Liu et al., 2016).

Smartphones have become the staple of the new era with the evolution of technology. However, the use of feature phones has not completely disappeared. Feature phones are still used in most places worldwide and are observed by some as a business devices. Therefore, the recommendation for feature phones in this study is still relevant and can contribute to the usability of fonts in feature phones. The information provided in Table 9 will help designers improve the already existing font size selection settings in most software applications by adding a degree of flexibility and control for older adults to select a suitable font size between specific ranges of font size for their age.

**Table 9:** Font size recommendations from selected paper and scenarios.

**Age range**	**Vision status**	**Device**	**Resolution/size**	**Font style**	**Recommended font size (points)**	**Scenario/use case**
51–78 years	All participants had 20/40 vision or better at a distance of 40.6 cm.	HP IPAQ	640 x 480 px	English characters	8–12 Points (Darroch et al., 2005)	The participants held the device at preferred viewing distances for the reading task.
57–70 years	With the assistance of visual aids (correction glasses, contact lenses).	IPhone 6, 5.5'	1,920 x 1080 px	Chinese characters	10.5 Points for information search 13 Points for intensive reading 13–14.5 Points for reading news, novels and instruction/ manuals. (Hou et al., 2020)	For reading tasks on mobile devices with Chinese characters. Viewing distance = (held at a comfortable distance)
66 Years	Normal vision or corrected to normal vision.	NEC mobile phones (NEC N6305)	480 x 320 px	Chinese characters	8 Points (Wang et al., 2009)	For reading tasks with Chinese inter-character spacing on a feature phone
60–89 years and older	For 16/20, corrected visual acuity or better. and regular reading aids	IPad	9.7-Inch	English character (Arial) and Japanese	14–22 Points (English character) 18 Points (Japanese) (42–66 arcminutes) (Lege et al., 2013; Yeh, 2015, 2020; Chatrangsan and Petrie, 2019)	For reading tasks on mobile devices Viewing distance = 40 cm

### Inconsistent evaluation parameters

The literature review results show pretty clearly the inconsistency in evaluation parameters, i.e., the factors considered in setting up the experiment and the experiment environment. Evaluation parameter in this context refers to the detailed information of the experiment environment and stimuli setup, i.e., viewing distance, font sizes, font typeface, visual angle of the font in arcminutes, visual acuity of subjects, device type, display size in inches and resolution. For example, it was observed that glare on display significantly affected viewing distance as the subjects had to either decrease or increase viewing distance to eliminate glare (Ko et al., 2014). These parameters are significant in evaluating reading speed/time, readability and legibility; therefore, researchers should endeavor to provide this information as they are relevant to the design field. From the reviewed literature, it was evident that these parameters provide concrete and detailed information on how the font size recommendations were made and the factors considered. Some studies investigating the reading speed/time of font, legibility and readability had varying experiment parameters, either failing to detail or omitting some of the mentioned parameters. This does not mean that the results obtained from these studies are not suitable. However, from a research and design standpoint, providing this specific information gives the designer or researcher concrete information to help in novel and innovative contributions to the field while speeding up the design and research process significantly.

### Uniform parameters to evaluate reading time/speed, readability and legibility

The parameter recommendation in this study results from data collected from the selected papers and the theoretical knowledge obtained from some of the papers on the psychophysics of reading. These parameters should be considered not just only for older adults but also for general users. These parameters include: viewing distances, font height (px, mm, pt), font typeface (serif or sans-serif), light source/ environmental lighting, display type, device type and resolution, visual acuity of subjects. The viewing distance refers to the distance between the eye and the stimuli. A visual acuity test is a visual test performed to evaluate the visual prowess of participants before an experiment. The implications and effect of these parameters on the resulting font size recommendation should be detailed in future studies. It is evident from the reviewed articles that these factors significantly influence results. This, together with the results from the eye-tracking test, provides practical information for UX designers and equips them with enough practical knowledge to improve usability, especially for the elderly.

### Uniformity of font size metric system

Font size is perceived by most in point or millimeter height on the screen (Ko et al., 2014). Some of the selected papers in this study provided the visual angle of the font in arcminutes, while others provided just font height in points and millimeters. According to Swearer (2011), Visual angle is a dimension used to indicate the size of visual stimuli subtended at the eye. The font's height in mm, pixel or points remains constant, but users do not have a constant viewing distance. For older adults, the normative age-related changes, especially in their eyesight, can significantly affect their viewing distance. Depending on the environmental lighting and the font type, reading time/speed, readability, and legibility can be significantly influenced. The visual angle of the font (VAF) is the ratio of font height (point, mm) to the viewing distance (VD). The researcher should note down the viewing distance of subjects, especially in cases where subjects are allowed to hold the device (if a mobile device) or stimuli at a preferred distance. Researchers could have a set viewing distance and compare the results to the self-selected viewing distance of the subjects to find any significant changes that could help other researchers and designers.

**Figure 5:**
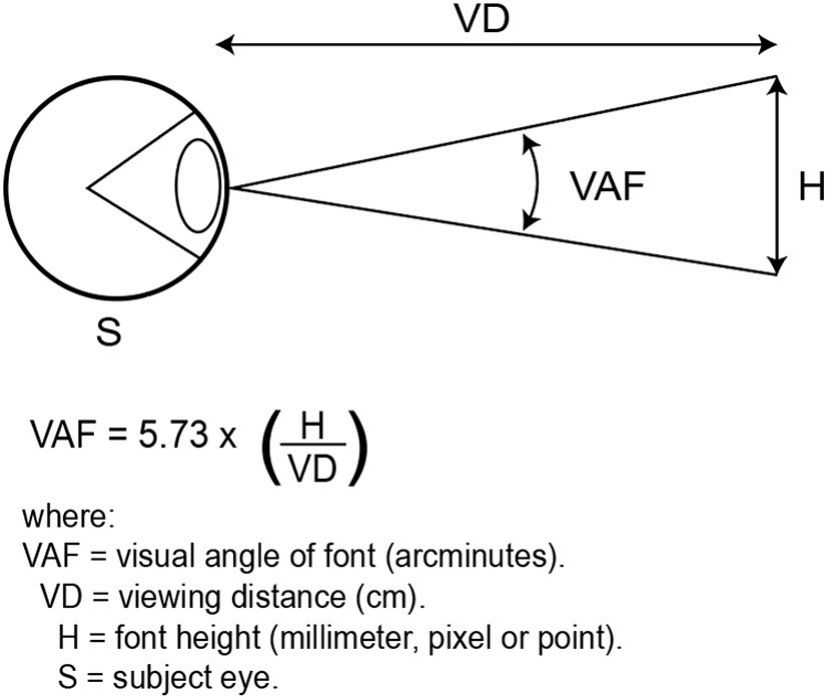
Schematic diagram for visual angle of font.

The importance of uniformly detailing the visual angle of the font is to provide information for vision scientists reliant on the angular size of the print; at the same time, typographers are reliant on the physical size of the print (Legge and Bigelow, 2011). Vision scientists prefer angular size to determine retinal image size (Legge and Bigelow, 2011). The schematic diagram of how to calculate the visual angle of font is shown in Figure 5.

Angular size in degrees is calculated as follows:


$$\begin{array}{l} VAF\;=5.73x\;\left(physical\;print/font\;size/viewingdistance\right). \end{array}$$

Typographers use the physical type size measure to determine how many characters per line, column, page, or screen of fixed dimensions (Legge and Bigelow, 2011). It is also used to estimate apparent character size at a typical viewing distance. The standard typographic measurement is the point. Table 10 shows the conversion between physical print/font size (point, mm, and inches) and visual angle (arcminutes):

**Table 10:** Conversion between units (Legge and Bigelow, 2011).

**Conversion between physical units**		
1. Conversion between		
Points and millimeters Points and inches	Point size = 2.66 x size in mm Point size = 72 x size in inches	Size in mm = 0.35 x point size Size in inches = 0.0138 x point size
2. Conversion to visual angle (VA) in degrees from physical print size (viewing distance of 40 cm = 16 inches). Conversion to visual angle (degrees) from		
Millimeters Points	VA = 0.143 x size in mm VA = 0.05 x size in point	
3. Conversion from a visual angle in degrees to physical print size in millimeters or points (viewing distance of 40 cm = 16 inches). Conversion from visual angle to		
Millimeters Points	Size in mm =7 x VA Size in points = 20 x VA	
4. Useful rules of thumb (viewing distance of 40 cm = 16 inches)		
1.4 mm = 4.0 point, subtends 0.20 degree 10 points = 3.5 mm, subtends 0.5 degree Visual angle in degrees = point size/20		

For a more in-depth understanding of the print/font size metrics and terminology, we recommend the article “Does print size matter for reading? A review of findings from vision science and typography” by Legge and Bigelow (2011).

### Eye-tracking as a usability evaluation method

In recent years, eye tracking has become a valuable means of evaluation for UX designers. The eye movement data help UX designers understand the users' mental model; the information makes it possible for most design flaws to be avoided. Eye-tracking can go a long way in helping UX designers and researchers understand the problems faced by older adults and not just with reading and visual search with intelligent devices but also in other aspects of life. This technology can significantly bridge the gap between the mental model of older users and designers, improve usability, and make it possible for precise usability recommendations.

Eye-tracking as an evaluation method is a valuable tool for obtaining valid eye movement data from user experience because it can tell us where the attention is distributed in interacting with mobile devices or computer devices (He et al., 2014). Eye movement measurements provide practical information for designers to help improve usability. For an in-depth discussion on eye tracking in usability evaluation and metrics, we recommend the books “Developing the Usability Testing Protocol” by Geisen and Romano Bergstrom (2017), “Eye tracking in user experience” by He et al. (2014) and “Measuring the User Experience” by Tullis and Albert (2013). Eye movement measures or eye-tracking metrics, such as fixation duration, gaze duration, time to fixation, number of fixation, re-fixations, scan paths and pupillometry, can all describe users' interaction with smart devices (He et al., 2014). Eye fixation is the point where the eye rests during reading. People with fewer fixations during reading usually have a higher reading pace than those who make more frequent fixations (Ways, 2021). Fixation data collected with an eye tracker suggests that users do not look at essential texts or images we want them to see, indicating a design flaw as observed in a study (He et al., 2014). Therefore, concerning design, this could mean that the element is not noticeable enough to be perceived as “important” by the user.

On the other hand, eye tracking as a usability evaluation comes with some expectations. The normative change that comes with aging older adults is in different ways. However, for the use of eye-tracking, the focus will be emphasized on the vision of the elderly. Older adults exhibit poor visual prowess than younger adults, thereby presenting the need for correction glasses and contact lenses. As iterated as one of the required parameters suggested earlier, a visual acuity test should be performed on all participants to evaluate their visual prowess and determine if the participant should participate in the experiment. This is subjective to the researcher whether a participant with poor vision should participate in the experiment with or without their correction glasses. The new eye trackers can record eye data from subjects with glasses or contacts, as evident in a study by Hou et al. (2020).

When employing eye tracking as a usability method, it is essential to note the fixation duration and number of saccades made by participants. Fixation duration is the relative engagement with the Area of Interest (AOI). It is important to note that the greater the average fixation duration, the greater the level of engagement. A saccade is a quick, simultaneous movement of both eyes between two or more phases of fixation in the same direction. Older adults have longer fixation duration and make longer saccades during reading (Butler et al., 1999; Goldberg and Wichansky, 2003; Kemper and McDowd, 2006; Schwarz, 2006; Rayner et al., 2009, 2014). Therefore, a suggestion that older adults read slower than younger adults. However, it would be interesting to know how older adults fair against each other in similar experiments, especially in investigating the suitable font size. This practical data can only be obtained from an eye tracker. The importance of this technology paves the way for more innovative research and discovery and provides concrete data, especially in reading scenarios where multiple factors such as font typeface, word spacing, line spacing, and font sizes are investigated. Eye-tracking provides a direct insight into the user's experience, which speeds up research and precise design recommendation.

### Limitation of the study

There may be some threats to the validity of this study, despite the systematic, planned process to attain the utmost achievable accuracy and relevant objectivity:

- Conclusion validity: The research questions were deemed relevant for this study. However, some other relevant research questions may have been overlooked, thus threatening the conclusion's validity. The research questions in this study were conceived by two independent authors and a supervisor to alleviate the threat to relevance.
- Construct validity: The entirety of the research process was manual; the search string used may have excluded some relevant papers that would have been selected. An attempt to mitigate this threat was the application of PICO criteria, thus resulting in an effective search string that contains a relevant collection of articles.
- Internal validity: Internal validity deals with data extraction and evaluation. The task was divided among the authors. One author carried out the data extraction, while the other reviewed the final results for validity.
- External Validity: The conclusions drawn in this paper are within the context of this study. Therefore this threat is not present.

#### Implications for research and practice

This paper provides several contributions regarding the reading time/speed, readability and legibility of font sizes for older adults on mobile and computer devices. This area of research is still expanding. This is evident in the frequency of publication within the last decade. This study has contributed by extracting usability findings specifically for older adults. Some areas that need exploration have also been identified in this study:

- The adoption of usability models in the design of the evaluation process.
- Automation of usability evaluation methods.
- Adoption of uniformed parameters for evaluation.
- Adoption of uniform metric for font size (i.e., the use of “visual angle of the font in arcminutes”) Support for recommendations, especially for older adults.
- Long term usability evaluation methods to ensure consistency among future studies in this area.

## Conclusion

The growing usage of mobile phones by older adults has encouraged usability studies to be done in other to improve usability for older adults in ways that the normative age-related issues do not restrict usage. This study investigated the previous studies on font size usability for older adults regarding reading time/speed, readability and legibility, suggested usability evaluation and empirical methods deemed efficient for future studies and uniform parameters and metrics.

This paper has analyzed studies that performed reading time/speed, readability and legibility evaluation of font sizes for older adults on mobile phones. Our findings demonstrate that research on this topic started expanding in 2015, and research is therefore still growing. We identified the need to use automated evaluation tools, specifically an eye tracker, as most papers used only questionnaires or think-aloud protocol to evaluate font size readability and legibility. Inconsistencies in experiment parameters were observed, and we provided recommendations for uniformity across future research. This paper provides a uniform and reuseable empirical method for usability evaluation of font size for older adults, necessary parameters to consider, and a uniform metric system by which all font size recommendations should abide. The contributions made in this paper specifically cover the gap of inconsistencies in terms of factors that significantly influence results. The suggestion of an eye tracker as a usability evaluation method is a way to introduce future researchers to the possibilities and potential of eye-tracking technology in visual science and inclusive design. We believe that the information in this paper will significantly improve data validity, help identify specific reading problems and speed up innovation in user experience, especially for older adults.

## Data availability statement

The original contributions presented in the study are included in the article/supplementary material, further inquiries can be directed to the corresponding author.

## Author contributions

GH in charge of this study. UA wrote the draft. All authors contributed to the article and approved the submitted version.

## Funding

This study was supported by Philosophy and Social Science Planning Foundation of Zhejiang Province grant No. 22NDJC11Z.

## Conflict of interest

The authors declare that the research was conducted in the absence of any commercial or financial relationships that could be construed as a potential conflict of interest.

## Publisher's note

All claims expressed in this article are solely those of the authors and do not necessarily represent those of their affiliated organizations, or those of the publisher, the editors and the reviewers. Any product that may be evaluated in this article, or claim that may be made by its manufacturer, is not guaranteed or endorsed by the publisher.
